# Associations between epileptic seizures in pregnancy and adverse pregnancy outcomes: A systematic review and meta-analysis

**DOI:** 10.1371/journal.pmed.1004580

**Published:** 2025-10-31

**Authors:** Oladipupo Olalere, Saba Tariq, Olanike Ajijola, Min-Dee Koh, Katie Crabb, Amie Wilson, Anwesa Chatterjee, Mairead Black, Katie Morris, Matthew Bluett-Duncan, Emily Taylor, Sereena Raju, Fatima Junaid, Rebecca Bromley, Ngawai Moss, Marta Garcia-Finana, John Craig, Amanda Wood, Annalise Weckesser, Judith Dyson, Catherine Nelson-Piercy, Elaine Denny, Tracy Roberts, Rachel McNeill, Shakila Thangaratinam, John Allotey

**Affiliations:** 1 Department of Metabolism and Systems Science, College of Medicine and Health, University of Birmingham, Birmingham, United Kingdom; 2 National Institute for Health and Care Research (NIHR) Birmingham Biomedical Research Centre, and Midlands Patient Safety Research Collaboration, (PSRC), Birmingham, United Kingdom; 3 College of Medicine and Health, University of Birmingham, Birmingham, United Kingdom; 4 Liverpool School of Tropical Medicine, Liverpool, United Kingdom; 5 Faculty of Health, Education and Life Sciences, Birmingham City University, Birmingham, United Kingdom; 6 Aberdeen Centre for Women’s Health Research, University of Aberdeen, Aberdeen, Scotland, United Kingdom; 7 School of Health Sciences, University of Birmingham, Birmingham, United Kingdom; 8 Division of Neuroscience, University of Manchester, Manchester, United Kingdom; 9 Royal Free Hospital, Royal Free London NHS Foundation Trust, London, United Kingdom; 10 Katies Team, Patient and Public Representative, London, United Kingdom; 11 Department of Biostatistics, University of Liverpool, Liverpool, United Kingdom; 12 Belfast Health and Social Care Trust, Belfast, United Kingdom; 13 Murdoch Children’s Research Institute, Melbourne, Victoria, Australia; 14 Women’s Health Academic Centre, Guy’s and St Thomas’ National Health Service Foundation Trust, London, United Kingdom; 15 Department of Applied Health Science, College of Medical and Health, University of Birmingham, Birmingham, United Kingdom; 16 Institute of Life Course and Medical Sciences, University of Liverpool, Liverpool, United Kingdom; 17 Liverpool Women’s NHS Foundation Trust, Liverpool, United Kingdom; 18 NIHR Applied Research Collaboration Northwest Coast, Liverpool, United Kingdom; University of Leeds, UNITED KINGDOM OF GREAT BRITAIN AND NORTHERN IRELAND

## Abstract

**Background:**

Epileptic seizures during pregnancy may increase the risk of adverse pregnancy outcomes. Socioeconomic disparities in epilepsy incidence may extend to seizure control. We conducted a systematic review and meta-analysis to assess the association between epileptic seizures during pregnancy and adverse pregnancy outcomes. We also evaluated the association between socioeconomic and individual-level factors and seizure occurrence.

**Methods and findings:**

We searched MEDLINE, Embase, CINAHL, and PsycINFO databases from inception to May 2025 for observational studies on pregnant women with epileptic seizures. We compared maternal and foetal outcomes in pregnant women with and without seizures and assessed the association between seizure occurrence and socioeconomic or individual-level factors. We used the Newcastle–Ottawa Scale to assess the risk of bias of included studies. Meta-analyses using random effects model were performed to estimate pooled odds ratios (ORs) with 95% confidence intervals (CIs).

From 13,381 identified publications, 25 studies (24,596 pregnancies) are included in this analysis. In pregnant women with epilepsy, women with seizures compared to those without had increased odds of caesarean birth (OR 1.62, 95% CI 1.14 to 2.30, *p* = 0.007), peripartum depression (OR 2.20, 95% CI 1.04 to 4.65, *p* = 0.04), and small for gestational age baby (OR 1.32, 95% CI 1.03 to 1.69, *p* = 0.03). The odds of preterm birth (OR 1.66, 95% CI 1.29 to 2.15, *p* < 0.001), low birthweight (OR 1.47, 95% CI 1.12 to 1.93, *p* = 0.006), and small for gestational age baby (OR 1.44, 95% CI 1.19 to 1.74, *p* < 0.001) were higher in women with seizures compared to women without epilepsy. The risk of seizures was greater in pregnant women with epilepsy with low income compared to those with higher income (OR 1.57, 95% CI 1.22 to 2.02, *p* < 0.001), and in women with focal epilepsy compared to those with generalised epilepsy (OR 1.84, 95% CI 1.54 to 2.20, *p* < 0.001). The number of studies for some outcomes was small, limiting subgroup analyses and detection of heterogeneity.

**Conclusion:**

Epileptic seizures are associated with increased risks of adverse maternal and foetal outcomes. Risk assessment to identify women with epilepsy at highest risk of seizures is needed to optimise care.

## Author summary

**Why was this study done?:**

- Epilepsy is one of the most common neurological disorders in women of childbearing age, and seizures during pregnancy are a leading cause of maternal death.
- Previous research primarily focussed on the effects of epilepsy and anti-seizure medication exposure on pregnancy outcomes, but the risks associated with epileptic seizures during pregnancy have not been quantified.
- There is limited understanding of how socioeconomic and individual-level maternal factors influence the risk of seizures in pregnant women with epilepsy.

**What did the researchers do and find?:**

- We conducted a systematic review and meta-analysis comparing outcomes among pregnant women with epilepsy who experienced seizures, to those who did not experience seizures and to women without epilepsy.
- We found that women with epileptic seizures during pregnancy had increased risks of adverse outcomes, including caesarean birth, preterm birth, low birth weight, small for gestational age babies, and peripartum depression.
- Women with epilepsy from low-income backgrounds and those with focal epilepsy were at greater risks of seizures during pregnancy.

**What do these findings mean?:**

- Epileptic seizures during pregnancy increase risks for both mother and baby.
- Income level and seizure type are associated with risk of seizures among pregnant women with epilepsy.
- Optimising seizure control and addressing social inequalities in pregnant women with epilepsy may improve outcomes for mothers and their babies.
- However, this review is limited by the small number of studies available for some outcomes, variation in study quality, and differences in how exposures and outcomes were defined across studies.

## Abstract 3

In this systematic review and meta-analysis, Oladipupo Olalere and colleagues assess whether epileptic seizures during pregnancy are associated with higher risk of adverse outcomes for mother and infant.

## Introduction

Epilepsy is one of the commonest neurological disorders affecting women of childbearing age [1]. Women with epilepsy are 10 times more likely to die in pregnancy than those without the condition, and seizures are a common cause of death [2]. Reports from the *Mothers and Babies: Reducing Risk through Audits and Confidential Enquiries across the UK* (MBRRACE-UK), consistently highlight that socioeconomic disparities and individual-level factors influence healthcare access, medication adherence, and pregnancy outcomes, and are key contributors to maternal death in the United Kingdom [2]. This is of particular concern in pregnant women with epilepsy who have seen a doubling of the rates of Sudden Unexpected Death in Epilepsy between 2013−15 and 2019−21 in UK and Ireland [3].

Quantification of the risks associated with seizures during pregnancy on maternal and foetal outcomes are needed to optimise care and management, balancing maternal seizure control with potential pregnancy risks [4,5]. There are known socioeconomic and individual-level inequalities in epilepsy incidence, healthcare access, and outcomes in the general population, but whether these inequalities also extends to occurrence of seizures in pregnancy is unknown [6,7]. Existing systematic reviews and meta-analyses on maternal epilepsy have focussed mainly on associations between epilepsy and antiseizure medication exposure and adverse pregnancy outcomes [8,9]. Despite the recognised clinical importance of preventing seizures in pregnancy [4], there is no quantifiable evidence on the association of epileptic seizures during pregnancy on maternal and foetal outcomes, nor on socioeconomic and individual-level factors.

We conducted a systematic review and meta-analysis to provide reliable estimates of the association between epileptic seizure occurrence during pregnancy and maternal and foetal outcomes, and to evaluate the association between socioeconomic and individual-level factors and seizures in pregnancy.

## Methods

We conducted this review using a prospective protocol registered with PROSPERO (ID CRD42023446520). This paper addresses objectives 5 and 6 of the PROSPERO registration and is reported in line with recommendations of the Preferred Reporting Items for Systematic Reviews and Meta-Analyses (PRISMA) guidelines [10] (S1 Appendix. PRISMA checklist).

### Search strategy and study selection

We searched MEDLINE (Ovid), Embase (Ovid), CINAHL, and PsycINFO (database inception through 21st May 2025) (S2 Appendix). Our search combined terms for pregnancy, obstetric, epilepsy, seizures, epileptic seizures, pregnancy disorders, adverse pregnancy outcome, and obstetric complications. Search results were imported into Covidence [11] and duplicates removed. Two reviewers (OO and OA) independently selected studies using a two-stage process. The first stage involved title and abstract screening, followed by full-text assessment of selected studies to determine eligibility. Disagreements were resolved by consensus.

We placed no restrictions on language and included all observational studies that reported maternal and foetal outcomes in the antenatal, intrapartum, or postnatal period in pregnant women with epilepsy who had seizures during pregnancy compared to those who did not or to pregnant women with no history of epilepsy. The following maternal outcomes were assessed: caesarean birth, induction of labour, gestational diabetes, antepartum haemorrhage, preeclampsia, spontaneous miscarriage, termination of pregnancy, gestational hypertension, postpartum haemorrhage, placental abruption, anaemia, premature rupture of membranes, and peripartum depression. Foetal outcomes included foetal growth restriction, small for gestational age, preterm birth (<37 weeks gestation), low birthweight (birthweight of less than 2.5 kg), perinatal death, 5-min Apgar scores less than 7, and any congenital anomaly.

We also included studies that reported socioeconomic (education status, income level, and employment status) or individual-level (ethnicity, maternal age, marital status, pregnancy planning, and epilepsy type) factors, in relation to seizure occurrence in pregnancy, to assess differences in seizure risk across these characteristics. We excluded case series, case reports, in vitro studies, books, editorials, comments and responses to comments, opinion pieces, posters, conference abstracts, animal studies, studies reporting non epileptic seizures, studies where epileptic seizure in pregnancy was not an exposure, or where no relevant data were provided.

### Quality assessment and data extraction

We used the Newcastle–Ottawa Scale [12] to assess the risk of bias and methodological quality of individual study. The scale evaluates studies across three domains: selection (maximum of or 4 stars), comparability (maximum of 2 stars), and ascertainment of outcome (maximum of 3 stars), with a total maximum of nine stars. Two reviewers (OO and MK) independently carried out quality assessment and allocated stars for adherence to the prespecified criteria. We considered a study as having a low risk of bias if it was given a total score of nine stars. Studies awarded six to eight stars with at least one star in the comparability domain were considered as having medium risk of bias, while all others were regarded as having high risk of bias.

Data were extracted in duplicates (OO and KC) using a predesigned and piloted data extraction form. Disagreements were resolved by consensus. Dichotomous data were extracted. We also extracted data on study characteristics such as year of study, country, study design, study aim, inclusion and exclusion criteria, details of comparator groups, and reported outcomes.

### Statistical analysis

We compared the odds of adverse maternal and foetal outcomes in pregnant women in the following groups: women with seizures versus women with epilepsy without seizures, women with seizures versus women with no history of epilepsy, and focal versus generalised onset seizures in women with epilepsy. We also compared the odds of epileptic seizures during pregnancy in women with epilepsy across the following socioeconomic factors: education level (primary versus secondary and above), income level (low versus moderate-to-high), and employment status (unemployed versus employed), and the following individual-level factors: maternal age based on commonly used groupings in obstetric research (24–34 years versus <24 years, 24–34 years versus >34 years), marital status (married/partnered versus single), pregnancy planning (planned versus unplanned pregnancies), and epilepsy type (focal versus generalised epilepsy). We reported the results obtained after pooling individual study estimates using random effects meta-analysis as odds ratio (OR) with 95% confidence intervals (CIs). A random effect was applied at the study level. Heterogeneity was summarised using the *I*^2^ statistic. We used Review Manager version 5 for analysis [13].

### Role of the funding source

The funder had no role in data collection, analysis, interpretation, report writing, or the decision to submit this report for publication. The corresponding author had full access to all the data in the study and had final responsibility for the decision to submit for publication.

## Results

**Fig 1:**
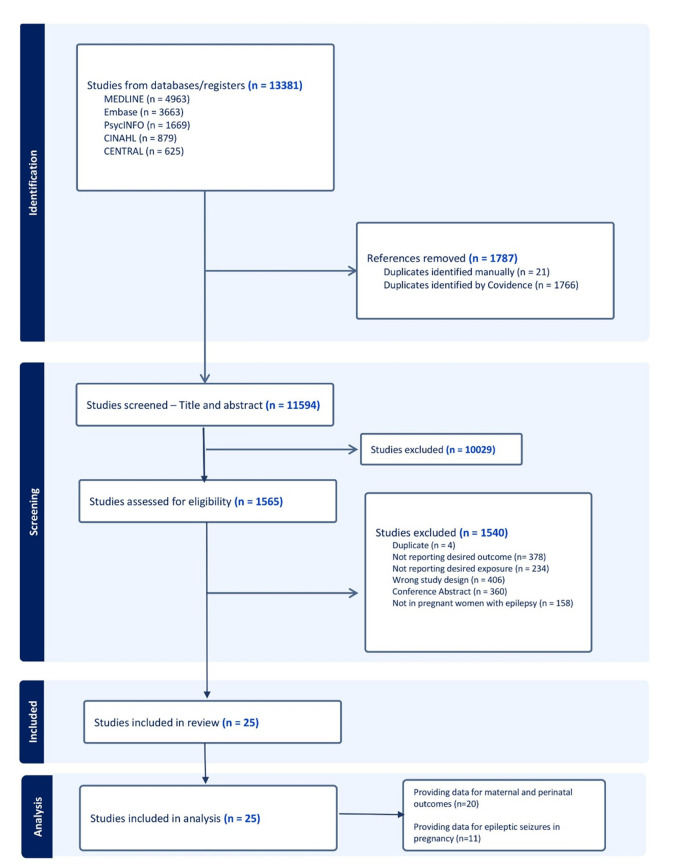
PRISMA 2020 flow diagram illustrating the study selection process for the systematic review and meta-analysis.

Of the 13,381 identified publications, 25 (24,596 pregnancies) met our inclusion criteria and were included in our analysis (Fig 1).

### Characteristics of the included studies

There were 13 retrospective [14–26] and 11 prospective [27–37] cohort studies, and one cross-sectional study [38]. Fourteen of the included studies were conducted in high income countries including USA [30,35], Australia [20,23,24], Canada [18,31], Japan [21], Iceland [22], China [17,26], Taiwan [38], Poland [37], and Spain [28], while the remaining 11 studies were conducted in India [19,33,36], Azerbaijan [14,32], Turkey [16,27], Nigeria [34], Thailand [15], Brazil [25], and Egypt [29]. Studies were published between 1998 and 2024. One study was published before 2000 [22], five studies between 2001 and 2010 [18,23,35,36,38], and 19 studies after 2010 [1,14–17,19–21,24–29,31–34,37]. Eight studies [16,19,21,23,24,27,30,38] adjusted for confounders including ethnicity, maternal age, mother’s level of education, household income, marital status, pregnancy intention, gestational age at enrolment, duration of epilepsy, and antiseizure medication use. Definitions varied across included studies, both for epilepsy and seizure occurrence. In the latter case, some studies counted only disabling seizures [31] or seizures requiring hospitalisation or treatment in the emergency department during [38], while others included all seizure types during pregnancy. One study [14] defined new-onset epilepsy during pregnancy as at least two unprovoked seizures occurring >24 h apart.

Eighteen studies (9,653 pregnancies) reported on maternal and foetal outcomes for pregnant women with seizures compared to those with epilepsy who had no seizures. Two studies (11,225 pregnancies) provided relevant outcome data for pregnant women with seizures compared to pregnant women without epilepsy. Thirteen studies (5,984 pregnancies) provided relevant data for women with focal onset seizures compare to those with generalised onset seizure. Nine studies (3,520 pregnancies) provided seizure rates across socioeconomic and sociodemographic factors (S3 Appendix).

**Fig 2:**
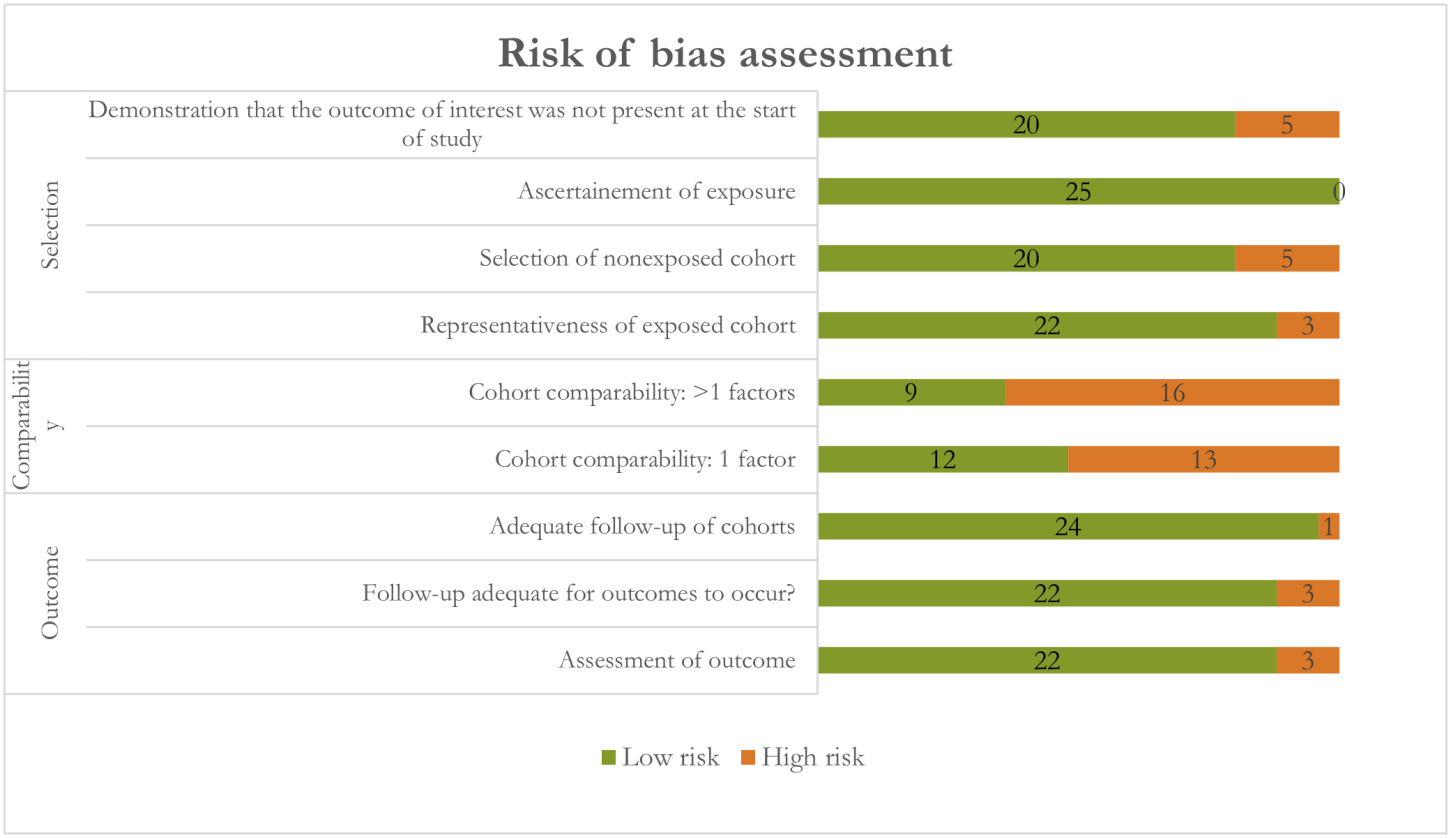
Risk of bias assessment of included studies using the Newcastle–Ottawa Scale.

Quality assessment by the Newcastle–Ottawa Scale showed that six studies (24%) had an overall low risk of bias, six (24%) had medium risk, and 13 (52%) had high risk of bias. Twenty studies (80%, 20/25) had low risk of bias in the selection domain, 36% (9/25) in the comparability domain, and 88% (22/25) in the outcome domain. (Fig 2 and S4 Appendix).

### Association between seizures and maternal and foetal outcomes

#### Compared to women with epilepsy with no seizures.

Maternal outcomes were assessed in 12 studies (4,544 pregnancies). Women with seizures compared to those without had increased odds of caesarean birth (OR 1.62, 95% CI 1.14 to 2.30, *p* = 0.007) and peripartum depression (OR 2.20, 95% CI 1.04 to 4.65, *p* = 0.04). The odds of spontaneous miscarriage were lower among women with seizures than women without (OR 0.42, 95% CI 0.29 to 0.61, *p* < 0.001). No differences were reported between the two groups for other maternal outcomes, including hypertensive disorders in pregnancy, induced labour, antepartum haemorrhage, postpartum haemorrhage, anaemia, gestational diabetes mellitus, premature rupture of membranes, placenta abnormality, uterine atony, and termination of pregnancy (Table 1 and S5 Appendix).

**Table 1:** Association between seizures and pregnancy outcomes in women with epilepsy.

Outcome	Studies (*n*)	Events (*n*)	Pregnancies (*n*)	Odds ratio (95% CI)	*I*^2^ (%)
Maternal outcomes					
**Spontaneous miscarriage**	**4**	**157**	**2,294**	**0.42 [0.29, 0.61]**	**0%**
**Caesarean birth**	**7**	**872**	**1,927**	**1.62 [1.14, 2.30]**	**43%**
Hypertensive disorder in pregnancy	4	85	762	1.04 [0.56, 1.92]	17%
Induced labour	3	148	647	1.16 [0.72, 1.87]	0%
Antepartum haemorrhage	2	53	533	0.99 [0.47, 2.10]	0%
Postpartum haemorrhage	4	74	456	1.39 [0.81, 2.39]	0%
**Peripartum depression**	**2**	**38**	**387**	**2.20 [1.04, 4.65]**	**0%**
Premature rupture of membrane	3	23	343	0.92 [0.20, 4.23]	46%
Induced abortion	3	12	307	2.36 [0.62, 9.03]	0%
Anaemia	2	50	229	1.41 [0.73, 2.69]	0%
Gestational Diabetes Mellitus	1	9	154	0.98 [0.25, 3.82]	–
Placenta abnormality	1	3	154	2.51 [0.22, 28.25]	–
Uterine atony	1	18	154	1.26 [0.42, 3.78]	–
Foetal outcomes					
Low birthweight	4	163	1,623	1.26 [0.91, 1.76]	0%
Preterm birth <37 weeks	8	272	2,342	1.18 [0.88, 1.59]	7%
**Small for gestational age**	**4**	**315**	**1,696**	**1.32 [1.03, 1.69]**	**0%**
Congenital anomaly	7	146	2,015	1.23 [0.77, 1.97]	21%
Apgar score <7 at 5 min	2	36	259	2.37 [0.41, 13.59]	65%
Perinatal death	4	80	2,946	0.63 [0.39, 1.00]	0%

Foetal and neonatal outcomes were assessed in 14 studies (6,285 pregnancies). The odds of babies being small-for-gestational age were increased in women who had seizures compared to those who did not (OR 1.32, 95% CI 1.03 to 1.69, *p* = 0.03). No differences were observed for other foetal outcomes (Table 1 and S5 Appendix).

#### Compared to women without epilepsy.

Two studies (11,225 pregnancies) explored the association between women with seizures compared to those without epilepsy and foetal outcomes. The odds of preterm birth <37 weeks (OR 1.66, 95% CI 1.29 to 2.15, *p* < 0.001), low birthweight (OR of 1.47, 95% CI 1.12 to 1.93, *p* = 0.006) and small-for-gestational age babies (OR 1.44, 95% CI 1.19 to 1.74, *p* < 0.001) were higher in pregnant women with seizures compared to pregnant women without epilepsy (Table 2 and S5 Appendix).

**Table 2:** Foetal outcomes in pregnant women with seizures vs. women without epilepsy.

Outcome	Studies (*n*)	Events (*n*)	Pregnancies (*n*)	Odds ratio (95% CI)	*I*^2^ (%)
Low birth weight	2	729	11,225	1.47 [1.12, 1.93]	0%
Preterm birth	2	765	11,225	1.66 [1.29, 2.15]	0%
Small for gestational age	2	1,902	11,225	1.44 [1.19, 1.74]	0%

#### Focal onset versus generalised onset seizures.

Thirteen studies (5,984 pregnancies) assessed the association between women with focal onset seizures and adverse maternal and foetal outcomes, compared to women with generalised onset seizures. Preterm birth <37 weeks was increased in women with focal seizures compared to those with generalised seizures (OR 4.69, 95% CI 1.07 to 20.57, *p* = 0.04). No differences were found between the two groups in odds of miscarriage, congenital anomaly, peripartum depression, premature rupture of membrane, caesarean birth, anaemia, hypertensive disorder in pregnancy, postpartum haemorrhage, and induced abortion (Table 3 and S5 Appendix).

**Table 3:** Pregnancy outcomes in women with focal onset seizures compared to those with generalised onset seizures.

Outcome	Studies (*n*)	Events (*n*)	Pregnancies (*n*)	Odds ratio (95% CI)	*I*^2^ (%)
Miscarriage	2	142	2074	1.27 [0.89, 1.81]	0%
Congenital anomaly	3	72	879	0.82 [0.47, 1.43]	0%
Peripartum depression	1	23	303	0.79 [0.33, 1.88]	–
Premature rupture of membrane	2	13	121	1.06 [0.27, 4.14]	0%
Caesarean birth	2	96	121	2.36 [0.46, 12.24]	47%
**Preterm birth <37 weeks**	**2**	**17**	**121**	**4.69 [1.07, 20.57]**	0%
Anaemia	1	16	41	1.19 [0.34, 4.19]	–
Hypertensive disorder in pregnancy	1	11	41	3.05 [0.67, 13.77]	–
Postpartum haemorrhage	1	17	41	0.63 [0.18, 2.22]	–
Induced abortion	1	4	87	1.15 [0.11, 11.63]	–

### Socioeconomic and individual-level factors associated with seizures in pregnant women with epilepsy

**Fig 3:**
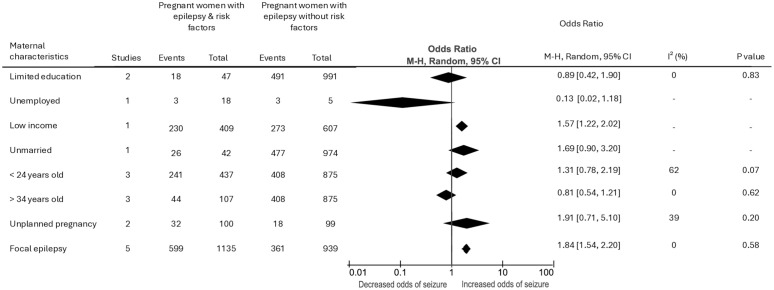
Forest plots of the association between socioeconomic and individual-level factors and seizures during pregnancy.

Evidence from one study showed greater risk of seizure in women with low income compared to those with higher income (OR 1.57, 95% CI 1.22 to 2.02, *p* < 0.001). Women with focal epilepsy had significantly increased odds of experiencing seizures during pregnancy compared to those with generalised epilepsy (OR 1.84, 95% CI 1.54 to 2.20, *p* < 0.001). Our analysis did not show any differences between other socioeconomic or individual-level factors and seizures in pregnancy (Fig 3 and S5 Appendix).

## Discussion

Our meta-analysis provides quantitative estimates of the magnitude of association between seizures in pregnancy and various maternal and foetal outcomes. Seizures in pregnant women with epilepsy were associated with a 2-fold increased risk of peripartum depression and a 62% increase in odds of caesarean section. The odds of low birthweight, preterm birth, and small-for-gestational age babies were increased in women with seizures during pregnancy compared to both women with epilepsy but no seizures and women without epilepsy, with higher odds observed in the comparison to women without epilepsy. Risk of preterm birth was increased 4-folds in women with focal onset seizures compared to those with generalised onset seizures. We found evidence of socioeconomic disparities in seizure occurrence during pregnancy, with higher risk in pregnant women with epilepsy with low income compared to those with higher income. Seizure risk was also higher in women with focal epilepsy compared to those with generalised epilepsy. Women with seizures in pregnancy had a lower risk of spontaneous miscarriage than those who were seizure-free.

To our knowledge, this is the only review and meta-analysis to assess the association between seizure in pregnancy, maternal and foetal outcomes, and individual-level socioeconomic factors. We carried out a comprehensive literature search without limitation, and our study was guided by a prospectively registered protocol. As participants were identified and reported as women in the included studies, we have used the term ‘pregnant women’ throughout to reflect the data as collected. Our various comparative analyses allowed us to comprehensively assess the association between seizures in pregnancy, seizure classifications, and adverse pregnancy outcomes. We were also able to assess the association between factors of socioeconomic inequalities and seizures in pregnant women with epilepsy.

Our systematic review has limitations. The studies varied in their definition of epilepsy, seizure occurrence, and outcomes, and important outcomes such as maternal mortality were not reported. Included studies span a period of more than 25 years, and it is possible that improvements in the management of pregnant women with epilepsy over this timespan could have affected seizure occurrence between studies. Studies also varied in whether and which confounders were adjusted, thus potentially affecting the interpretations of our findings. However, some factors such as individual lifestyles and irregular medication use, may act as mediators rather than confounders of the relationship between seizures and pregnancy outcome, and adjusting for these may obscure the pathways through which seizures in pregnancy affect maternal and foetal outcomes [39]. Recognising these mediators is essential to understanding the full complexity of how seizures influence health outcomes during pregnancy. Evidence to provide reliable estimates for some adverse outcomes in our review was limited due to the small number of studies and participants, and we were unable to carry out subgroup analyses, such as by anti-seizure medication, on adverse outcomes. The small number of eligible studies contributing to individual analyses also limited our ability to assess temporal trends or publication bias and explains why many of our meta-analyses resulted in *I*^2^ values of 0%. However, our meta-analyses synthesise available evidence, making the best use of existing data, and represents a step towards understanding the complex relationship between seizures in pregnancy, socioeconomic and individual-level factors and pregnancy outcomes.

While previous studies have reported increased risks of adverse maternal and foetal outcomes in women with epilepsy compared to those without [8,9], our findings specifically focus on the association between seizures during pregnancy and maternal and foetal outcomes. We found that seizures in pregnancy are predictors of poorer outcomes in pregnant women with epilepsy. This is important as it underscores the elevated risks associated with seizures during pregnancy, and not just the underlying epilepsy.

Consistent with earlier meta-analyses reporting increased risk of caesarean birth in women with epilepsy [8,9], our study found higher risks of caesarean birth in pregnant women with epilepsy who had seizures compared to those without seizures. While our study was not designed to assess the clinical appropriateness of these decision, the observed rates may reflect the clinical complexities and risks associated with seizure activity during labour and delivery [4]. Rates of caesarean section vary by healthcare setting, provider expertise, and access to specialist services, which may also influence clinical decision-making and contribute to the variations in caesarean rates observed across studies in this review [40]. The pattern of increasing risk of low birthweight, preterm birth, and small-for-gestational-age babies observed in women with epilepsy with seizures compared to those with epilepsy but no seizures, and to pregnant women without epilepsy, indicates that the presence of seizures during pregnancy increases the likelihood of these adverse foetal outcomes. The observed increase in peripartum depression in women with seizures aligns with other reviews that found patients with epilepsy at higher risk of major depressive disorders [41,42], with pregnancy and postpartum periods being windows of increased vulnerability [30]. This heightened risk may be attributed to several factors, including the psychological burden of managing a chronic condition like epilepsy, the physical effects of seizures, and the stress of adjusting to the challenges of caring for a newborn [43]. Increased seizure frequency may also reflect greater epilepsy severity, which could be associated with a higher risk of depression. However, information on seizure frequency and epilepsy severity was not assessed in this review.

The body of research examining the intersection between socioeconomic and individual-level factors on seizure occurrence and pregnancy outcomes in women with epilepsy is limited. Most studies [8,9,17,27] have focussed broadly on epilepsy without differentiating between seizure types or considering the full spectrum of factors that may affect outcomes. Evidence from one study in our review showed that women from lower-income backgrounds may have a higher risk of seizure during pregnancy compared to those with higher income [38]. This retrospective study from Taiwan included 1,016 women with epilepsy, and while it adjusted for confounders, its findings may reflect factors specific to that setting. Socioeconomic status is a known determinant of general health outcomes [44,45], but evidence for its association with seizure occurrence in pregnancy remains limited and should therefore be interpreted with caution.

In the UK, disparities in access can exist due to factors such as geographical location, availability of specialised services, or variations in healthcare providers’ knowledge about managing epilepsy in pregnancy [46]. Women from lower socioeconomic backgrounds may also face greater psychosocial stressors, such as financial instability, housing insecurity, or difficulties in accessing mental health support, which may contribute to poorer seizure control. Seizure activity, particularly when poorly controlled, may also affect socioeconomic circumstances through its impact on employment, income, or access to care [47]. This bidirectional relationship likely applies beyond pregnancy [48]. Women with focal epilepsy were at greater risk of seizures and preterm birth compared to those with generalised epilepsy; however, the finding for preterm birth is based on just two studies and should be interpreted cautiously. Existing studies show that focal seizures may be more difficult to control and more likely to be associated with adverse outcomes [49–51].

The observed lower miscarriage risk in women with seizures during pregnancy may reflect selection bias or confounding in the available data. While polytherapy is associated with increased risk of miscarriage [20,52], and used in women with more refractory epilepsy [53,54], the relationship between medication use, seizure control, and miscarriage risk is likely complex and incompletely captured in the included studies. Variability in miscarriage reporting across studies may have also contributed to these findings, particularly if early losses were more likely to go unreported among women with less well-managed epilepsy. Women who experience seizures during pregnancy often receive more intensive obstetric and neurological monitoring, which can lead to earlier identification and management of complications, potentially influencing outcomes.

Preconception care is an important opportunity to optimise seizure control and overall health before pregnancy. Women with well-controlled epilepsy are less likely to experience seizures during pregnancy [55], and in our study, seizure occurrence was associated with increased risk of adverse outcomes. Healthcare providers should prioritise discussions around medication adherence during preconception counselling, to ensure that women understand the balance between seizure control and potential medication risks [45]. Women with focal seizures may require increased monitoring from preconception care through the postpartum period, and healthcare providers should be aware of the increased risk of seizures during pregnancy in these women.

Our findings that income level and seizure type influence seizure risk highlight both the social and clinical factors that shape epilepsy outcomes. Women from lower-income backgrounds or those with complex social circumstances may require additional support to effectively manage epilepsy during pregnancy. Targeted efforts to reach these high-risk groups, are necessary to ensure equitable outcomes. The evidence linking income level to seizure occurrence in pregnancy comes from a single study in Taiwan and requires replication in other settings. Larger, well-designed studies are needed to better quantify trimester-specific risks and to assess the implications of seizures during pregnancy on maternal and child health in the short- and long term. Developing, implementing, and evaluating a risk assessment tool specifically for seizures in pregnancy could significantly enhance clinical practice [56–59]. Such a tool would enable healthcare providers to identify women at higher risk of seizures based on individual clinical and socioeconomic factors, facilitating early intervention and tailored management strategies.

## Supporting information

S1 Appendix PRISMA checklist for reporting systematic reviews and meta-analyses. This checklist is reproduced from Page MJ, McKenzie JE, Bossuyt PM, et al. The PRISMA 2020 statement: an updated guideline for reporting systematic reviews. *BMJ* 2021;372:n71, https://www.bmj.com/content/372/bmj.n71
(DOCX)

S2 Appendix Search terms and strategy. (DOCX)

S3 Appendix Characteristics of included studies. (DOCX)

S4 Appendix Risk of bias assessment. (DOCX)

S5 Appendix Forest plots of individual analyses. (DOCX)

S6 Appendix Data extraction sheet. (DOCX)

## Abbreviations

CI: confidence interval
OR: odds ratio
PRISMA: Preferred Reporting Items for Systematic Reviews and Meta-Analyses
