# Supplementary material for: Associations between epileptic seizures in pregnancy and adverse pregnancy outcomes: A systematic review and meta-analysis
Source: PLoS Med. 2025 Oct 31;22(10):e1004580. doi: 10.1371/journal.pmed.1004580 (PMC12578136; doi:10.1371/journal.pmed.1004580)
Supplement: S2 Appendix — (DOCX) [file pmed.1004580.s002.docx]

**S2 Appendix. Search terms and strategy**

Ovid MEDLINE(R) ALL <1946 to July 14, 2024>

1 exp Pregnancy/ 1008739

2 exp Pregnant Women/ 14625

3 (pregnan* or obstetric* or wom* or gravid*).ab,kf,kw,ti,tw. 1813305

4 1 or 2 or 3 2250404

5 exp Epilepsy/ 126209

6 exp Epileptic Syndromes/ 28772

7 (epilepsy or epileptic*).ab,kf,kw,ti,tw. 157983

8 5 or 6 or 7 186485

9 exp Pregnancy Outcome/ 86050

10 (pregnan* outcome* or adverse pregnan* outcome* or obstetric* complicat* or pregnan* disorder* or obstetric* outcome*).ab,kf,kw,ti,tw. 42189

11 (neurodevelopment* or neurodevelopment* disorder or developmental disability* or cognitive development* disorder* or dysparaxia or intellectual disabilit* or pervasive developmental disorder* or behavior* or behavior* disorder* or neurobehavior* disorder* or neurocognitive disorder* or development* regression or developmental coordination disorder* or psychomotor developmental disorder or language delay* or social impairment).ab,kf,kw,ti,tw. 1315711

12 exp Sudden Unexpected Death in Epilepsy/ 297

13 "Sudden unexpected death in epilepsy".ab,kw,ti,tw. 1264

14 exp Abortion, Spontaneous/ 38989

15 (miscarriage* or spontaneous abortion* or habitual abortion* or pregnancy loss* or f?etal loss* or f?etal death* or f?etal demise* or intrauterine loss* or intrauterine death* or intrauterine demise*).ab,kw,ti,tw. 51478

16 exp Fetal Death/ 31227

17 exp Infant, Low Birth Weight/ 38996

18 "birth weight*".ab,kw,ti,tw. 72126

19 exp Fetal Growth Retardation/ 18845

20 (IUGR or intra-uterine growth or intrauterine growth or f?etal growth or (f?etus adj3 growth)).ab,kw,ti,tw. 34100

21 exp Stillbirth/ or exp Infant Mortality/ 37170

22 "stillbirth*".ab,kw,ti,tw. 15472

23 ((infant or new born or newborn or neonatal) adj3 mortality).ab,kw,ti,tw. 26301

24 exp Intensive Care Units, Neonatal/ 18194

25 (NICU or neonatal intensive care unit).ab,kw,ti,tw. 25120

26 exp Infant, Small for Gestational Age/ 8633

27 small for gestational age.ab,kw,ti,tw. 13329

28 exp Apgar Score/ 8112

29 APGAR.ab,kw,ti,tw. 14558

30 ((f?etal or f?etus or infant* or neonatal* or newborn* or baby or babies) adj3 hypoglyc?emi*).ab,kw,ti,tw. 2982

31 exp Autistic Disorder/ 25379

32 (autism or autistic).ab,kw,ti,tw. 66308

33 exp Attention Deficit Disorder with Hyperactivity/ 34777

34 (attention deficit hyperactiv* disorder* or ADHD).ab,kw,ti,tw. 39864

35 exp Resuscitation/ and exp Infant, Newborn/ 12399

36 ((newborn* or neonatal* or infant*) adj3 resuscitation).ab,kw,ti,tw. 3877

37 exp Premature Birth/ 21354

38 ((preterm* or premature) adj3 (birth* or deliver* or infant* or newborn* or baby or babies or neonat*)).ab,kw,ti,tw. 108810

39 exp Diabetes, Gestational/ 17482

40 (gestational diabete* or "DIABETES IN PREGNANCY").ab,kw,ti,tw. 22051

41 ((HYPERTENSION or HYPERTENSIVE) adj3 PREGNAN*).ab,kw,ti,tw. 15637

42 exp Postpartum Hemorrhage/ 8494

43 POSTPARTUM H?EMORRHAGE.ab,kw,ti,tw. 7825

44 antepartum h?emorrhage.ab,kw,ti,tw. 977

45 exp Pregnancy Complications/ 475466

46 exp Obstetric Labor Complications/ 80313

47 (pregnancy complication* or labo?r complication*).ab,kw,ti,tw. 22756

48 exp Depression, Postpartum/ 7463

49 exp Postpartum Period/ and exp Anxiety/ 839

50 ((postpartum or postnatal) adj3 (depression or anxiety)).ab,kw,ti,tw. 9432

51 exp Fetal Membranes, Premature Rupture/ 8287

52 (premature rupture of membrane* or prelabo?r rupture of membrane*).ab,kw,ti,tw. 5631

53 exp Abruptio Placentae/ 2442

54 (placenta* adj3 abrupt*).ab,kw,ti,tw. 4332

55 exp Pregnancy, Ectopic/ 15529

56 ((ectopic or tubal) adj3 pregnan*).ab,kw,ti,tw. 13507

57 exp Breast Feeding/ 44058

58 (breast feeding or breast fed).ab,kw,ti,tw. 18484

59 exp Abortion, Induced/ 42885

60 (abortion* adj3 (induced or medical*)).ab,kw,ti,tw. 9081

61 (termination of pregnancy or pregnancy termination).ab,kw,ti,tw. 8961

62 exp Maternal Mortality/ or exp Maternal Death/ 11914

63 (maternal adj3 (death* or motalit*)).ab,kw,ti,tw. 10210

64 exp Cesarean Section/ 53247

65 (cesarean or caearean or cesarian or caearian or "c section" or C-section*).ab,kw,ti,tw. 50275

66 exp Extraction, Obstetrical/ 3636

67 ((obstetric* or mechanical or forcep* or vacuum) adj3 (deliver* or extraction* or birth*)).ab,kw,ti,tw. 8751

68 exp Obstetrical Forceps/ or exp Vacuum Extraction, Obstetrical/ 2813

69 ventouse.ab,kw,ti,tw. 275

70 exp Pre-Eclampsia/ or exp Eclampsia/ 38776

71 (eclampsia or preeclampsia or eclamptic or preeclamptic).ab,kw,ti,tw. 44143

72 exp Labor, Induced/ 10244

73 ((labour or labor or Birth or childbirth or deliver*) adj3 (induction or induce*)).ab,kw,ti,tw. 14668

74 exp Seizures/ 73859

75 (seizure* or convulsion*).ab,kw,ti,tw. 158706

76 exp Accidental Injuries/ 232

77 exp "Wounds and Injuries"/ 1011133

78 (injur* or wound* or trauma).ab,kw,ti,tw. 1360444

79 exp Hospitalization/ 292900

80 (hospitali?ation* or hospital admission* or (HOSPITAL adj3 admitted)).ab,kw,ti,tw. 305965

81 74 or 75 176838

82 76 or 78 1360484

83 77 or 78 1937021

84 79 or 80 506612

85 81 and 82 11203

86 81 and 83 13606

87 81 and 84 5065

88 9 or 10 or 11 or 12 or 13 or 14 or 15 or 16 or 17 or 18 or 19 or 20 or 21 or 22 or 23 or 24 or 25 or 26 or 27 or 28 or 29 or 30 or 31 or 32 or 33 or 34 or 35 or 36 or 37 or 38 or 39 or 40 or 41 or 42 or 43 or 44 or 45 or 46 or 47 or 48 or 49 or 50 or 51 or 52 or 53 or 54 or 55 or 56 or 57 or 58 or 59 or 60 or 61 or 62 or 63 or 64 or 65 or 66 or 67 or 68 or 69 or 70 or 71 or 72 or 73 or 85 or 86 or 87 2195492

89 4 and 8 and 88 4656

Embase <1974 to 2023 August 14>

1 exp pregnancy/ 772570

2 exp pregnant woman/ 111611

3 (pregnan* or obstetric* or wom* or gravid*).ab,kw,ti,tw. 2412342

4 1 or 2 or 3 2654231

5 exp epilepsy/ 271992

6 (epilepsy or epileptic*).ab,kw,ti,tw. 224175

7 5 or 6 314120

8 exp pregnancy outcome/ 81507

9 exp pregnancy complication/ 158203

10 exp labor complication/ 232737

11 (pregnan* outcome* or adverse pregnan* outcome* or obstetric* complicat* or pregnan* disorder* or obstetric* outcome* or pregnancy complication* or labo?r complication*).ab,kw,ti,tw. 75748

12 (neurodevelopment* or neurodevelopment* disorder or developmental disability* or cognitive development* disorder* or dysparaxia or intellectual disabilit* or pervasive developmental disorder* or behavior* or behavior* disorder* or neurobehavior* disorder* or neurocognitive disorder* or development* regression or developmental coordination disorder* or psychomotor developmental disorder or language delay* or social impairment).ab,kw,ti,tw. 1448216

13 exp autism/ 94478

14 (autism or autistic).ab,kw,ti,tw. 85459

15 exp attention deficit hyperactivity disorder/ 9509

16 (attention deficit hyperactiv* disorder* or ADHD).ab,kw,ti,tw. 56289

17 exp sudden unexpected death in epilepsy/ 420

18 Sudden unexpected death in epilepsy.ab,kw,ti,tw. 1815

19 exp fetus death/ 45944

20 exp low birth weight/ 60113

21 birth weight.ab,kw,ti,tw. 94700

22 exp intrauterine growth retardation/ 38966

23 (IUGR or intra-uterine growth or intrauterine growth or f?etal growth or (f?etus adj3 growth)).ab,kw,ti,tw. 49834

24 exp stillbirth/ 22996

25 exp infant mortality/ 21881

26 still birth.ab,kw,ti,tw. 1071

27 ((infant or new born or newborn or neonatal) adj3 mortality).ab,kw,ti,tw. 29575

28 exp newborn intensive care/ or exp neonatal intensive care unit/ 49245

29 (NICU or neonatal intensive care unit or newborn* intensive care unit).ab,kw,ti,tw. 39335

30 exp small for gestational age/ 1176

31 small for gestational age.ab,kw,ti,tw. 18345

32 exp Apgar score/ 32751

33 APGAR.ab,kw,ti,tw. 23064

34 ((f?etal or f?etus or infant* or neonatal* or newborn* or baby or babies) adj3 hypoglyc?emi*).ab,kw,ti,tw. 4437

35 resuscitation/ and infant/ 5952

36 ((newborn* or neonatal* or infant*) adj3 resuscitation).ab,kw,ti,tw. 4878

37 exp spontaneous abortion/ or exp abortion/ or exp medical abortion/ or exp induced abortion/ 118073

38 ((miscarriage* or spontaneous abortion* or habitual abortion* or pregnancy loss* or f?etal loss* or f?etal death* or f?etal demise* or intrauterine loss* or intrauterine death* or intrauterine demise* or abortion*) adj3 (induced or medical*)).ab,kw,ti,tw. 10334

39 exp prematurity/ 126077

40 ((preterm* or premature) adj3 (birth* or deliver* or infant* or newborn* or baby or babies or neonat*)).ab,kw,ti,tw. 145291

41 exp pregnancy diabetes mellitus/ 47042

42 (gestational diabete* or "DIABETES IN PREGNANCY").ab,kw,ti,tw. 34572

43 exp "eclampsia and preeclampsia"/ or exp eclampsia/ 74499

44 (eclampsia or preeclampsia or eclamptic or preeclamptic).ab,kw,ti,tw. 65201

45 ((HYPERTENSION or HYPERTENSIVE) adj3 PREGNAN*).ab,kw,ti,tw. 23115

46 exp postpartum hemorrhage/ 18442

47 POSTPARTUM H?EMORRHAGE.ab,kw,ti,tw. 12089

48 antepartum h?emorrhage.ab,kw,ti,tw. 1502

49 exp postnatal depression/ 7110

50 ((postpartum or postnatal) adj3 (depression or anxiety)).ab,kw,ti,tw. 12539

51 exp premature fetus membrane rupture/ 13216

52 (premature rupture of membrane* or prelabo?r rupture of membrane*).ab,kw,ti,tw. 8388

53 (placenta* adj3 abrupt*).ab,kw,ti,tw. 6588

54 exp ectopic pregnancy/ 22641

55 ((ectopic or tubal) adj3 pregnan*).ab,kw,ti,tw. 17105

56 exp breast feeding/ 66069

57 (breast feeding or breast fed).ab,kw,ti,tw. 22134

58 exp pregnancy termination/ 59303

59 (termination of pregnancy or pregnancy termination).ab,kw,ti,tw. 12491

60 exp maternal mortality/ or exp maternal death/ 27175

61 (maternal adj3 (death* or motalit*)).ab,kw,ti,tw. 13729

62 exp cesarean section/ 123721

63 (cesarean or caearean or cesarian or caearian or "c section" or C-section*).ab,kw,ti,tw. 69966

64 exp obstetric forceps/ 461

65 exp vacuum extraction/ or exp forceps delivery/ 5753

66 ((obstetric* or mechanical or forcep* or vacuum) adj3 (deliver* or extraction* or birth*)).ab,kw,ti,tw. 11893

67 ventouse.ab,kw,ti,tw. 542

68 exp labor induction/ 16219

69 ((labour or labor or Birth or childbirth or deliver*) adj3 (induction or induce*)).ab,kw,ti,tw. 20835

70 exp seizure/ 216845

71 (seizure* or convulsion*).ab,kw,ti,tw. 235607

72 exp accidental injury/ 4836

73 (injur* or wound* or trauma).ab,kw,ti,tw. 1764264

74 exp hospitalization/ 526139

75 (hospitali?ation* or hospital admission* or (HOSPITAL adj3 admitted)).ab,kw,ti,tw. 510997

76 70 or 71 305573

77 72 or 73 1765812

78 74 or 75 775875

79 76 and 77 22072

80 76 and 78 13212

81 8 or 9 or 10 or 11 or 12 or 13 or 14 or 15 or 16 or 17 or 18 or 19 or 20 or 21 or 22 or 23 or 24 or 25 or 26 or 27 or 28 or 29 or 30 or 31 or 32 or 33 or 34 or 35 or 36 or 37 or 38 or 39 or 40 or 41 or 42 or 43 or 44 or 45 or 46 or 47 or 48 or 49 or 50 or 51 or 52 or 53 or 54 or 55 or 56 or 57 or 58 or 59 or 60 or 61 or 62 or 63 or 64 or 65 or 66 or 67 or 68 or 69 or 79 or 80 2450824

82 4 and 7 and 81 7444

83 limit 82 to "remove medline records" 3663

Cochrane 15^th^ August 2023

#1 MeSH descriptor: [Pregnancy] explode all trees 31338

#2 MeSH descriptor: [Pregnant Women] explode all trees 764

#3 (pregnan* or obstetric* or wom* or gravid*):ti,ab,kw (Word variations have been searched) 227620

#4 #1 OR #2 OR #3 227806

#5 MeSH descriptor: [Epilepsy] explode all trees 3451

#6 (epilepsy or epileptic*):ti,ab,kw (Word variations have been searched) 9344

#7 #5 OR #6 9435

#8 MeSH descriptor: [Pregnancy Outcome] explode all trees 5138

#9 MeSH descriptor: [Pregnancy Complications] explode all trees 16069

#10 MeSH descriptor: [Obstetric Labor Complications] explode all trees 5270

#11 (pregnan* outcome* or adverse pregnan* outcome* or obstetric* complicat* or pregnan* disorder* or obstetric* outcome* or pregnancy complication* or labo?r complication*):ti,ab,kw (Word variations have been searched) 64340

#12 (neurodevelopment* or neurodevelopment* disorder or developmental disability* or cognitive development* disorder* or dysparaxia or intellectual disabilit* or pervasive developmental disorder* or behavior* or behavior* disorder* or neurobehavior* disorder* or neurocognitive disorder* or development* regression or developmental coordination disorder* or psychomotor developmental disorder or language delay* or social impairment):ti,ab,kw (Word variations have been searched) 147078

#13 MeSH descriptor: [Autistic Disorder] explode all trees 1394

#14 (AUTISM OR AUTISTIC):ti,ab,kw (Word variations have been searched) 5099

#15 MeSH descriptor: [Attention Deficit Disorder with Hyperactivity] explode all trees 3475

#16 (attention deficit hyperactiv* disorder* or ADHD):ti,ab,kw (Word variations have been searched) 7169

#17 MeSH descriptor: [Sudden Unexpected Death in Epilepsy] explode all trees 2

#18 (Sudden unexpected death in epilepsy):ti,ab,kw (Word variations have been searched) 32

#19 MeSH descriptor: [Aborted Fetus] explode all trees 0

#20 MeSH descriptor: [Fetal Mortality] explode all trees 2

#21 MeSH descriptor: [Infant, Low Birth Weight] explode all trees 2645

#22 ("birth weight"):ti,ab,kw (Word variations have been searched) 12747

#23 MeSH descriptor: [Fetal Growth Retardation] explode all trees 539

#24 (IUGR or intra-uterine growth or intrauterine growth or f?etal growth or (f?etus NEAR/3 growth)):ti,ab,kw (Word variations have been searched) 4594

#25 MeSH descriptor: [Infant Mortality] explode all trees 894

#26 ("still births"):ti,ab,kw (Word variations have been searched) 112

#27 ((infant or new born or newborn or neonatal) NEAR/3 mortality):ti,ab,kw (Word variations have been searched) 4245

#28 MeSH descriptor: [Intensive Care Units, Neonatal] explode all trees 1036

#29 (NICU or neonatal intensive care unit or newborn* intensive care unit):ti,ab,kw (Word variations have been searched) 7101

#30 MeSH descriptor: [Apgar Score] explode all trees 1017

#31 ("Apgar"):ti,ab,kw (Word variations have been searched) 5565

#32 ("small for date"):ti,ab,kw (Word variations have been searched) 565

#33 ((f?etal or f?etus or infant* or neonatal* or newborn* or baby or babies) NEAR/3 hypoglyc?emi*):ti,ab,kw (Word variations have been searched) 579

#34 ((newborn* or neonatal* or infant*) NEAR/3 resuscitation):ti,ab,kw (Word variations have been searched) 784

#35 MeSH descriptor: [Abortion, Induced] explode all trees 1358

#36 MeSH descriptor: [Abortion, Spontaneous] explode all trees 1209

#37 ((miscarriage* or spontaneous abortion* or habitual abortion* or pregnancy loss* or f?etal loss* or f?etal death* or f?etal demise* or intrauterine loss* or intrauterine death* or intrauterine demise* or abortion*) NEAR/3 (induced or medical*)):ti,ab,kw (Word variations have been searched) 8944

#38 MeSH descriptor: [Premature Birth] explode all trees 2121

#39 ((preterm* or premature) NEAR/3 (birth* or deliver* or infant* or newborn* or baby or babies or neonat*)):ti,ab,kw (Word variations have been searched) 19691

#40 MeSH descriptor: [Diabetes, Gestational] explode all trees 1395

#41 (gestational diabete* or "DIABETES IN PREGNANCY"):ti,ab,kw (Word variations have been searched) 4433

#42 MeSH descriptor: [Eclampsia] explode all trees 278

#43 MeSH descriptor: [Pre-Eclampsia] explode all trees 1549

#44 (eclampsia or preeclampsia or eclamptic or preeclamptic):ti,ab,kw (Word variations have been searched) 4495

#45 ((HYPERTENSION or HYPERTENSIVE) NEAR/3 PREGNAN*):ti,ab,kw (Word variations have been searched) 2120

#46 MeSH descriptor: [Postpartum Hemorrhage] explode all trees 972

#47 (POSTPARTUM H?EMORRHAGE):ti,ab,kw (Word variations have been searched) 2872

#48 (antepartum h?emorrhage):ti,ab,kw (Word variations have been searched) 191

#49 MeSH descriptor: [Depression, Postpartum] explode all trees 867

#50 ((postpartum or postnatal) NEAR/3 (depression or anxiety)):ti,ab,kw (Word variations have been searched) 2708

#51 MeSH descriptor: [Fetal Membranes, Premature Rupture] explode all trees 618

#52 (premature rupture of membrane* or prelabo?r rupture of membrane*):ti,ab,kw (Word variations have been searched) 1634

#53 MeSH descriptor: [Abruptio Placentae] explode all trees 40

#54 (placenta* NEAR/3 abrupt*):ti,ab,kw (Word variations have been searched) 491

#55 MeSH descriptor: [Pregnancy, Ectopic] explode all trees 223

#56 ((ectopic or tubal) NEAR/3 pregnan*):ti,ab,kw (Word variations have been searched) 1038

#57 MeSH descriptor: [Breast Feeding] explode all trees 2664

#58 (breast feeding or breast fed):ti,ab,kw (Word variations have been searched) 8091

#59 (termination of pregnancy or pregnancy termination):ti,ab,kw (Word variations have been searched) 2937

#60 MeSH descriptor: [Maternal Mortality] explode all trees 215

#61 MeSH descriptor: [Maternal Death] explode all trees 51

#62 (maternal NEAR/3 (death* or motalit*)):ti,ab,kw (Word variations have been searched) 827

#63 MeSH descriptor: [Cesarean Section] explode all trees 4652

#64 (cesarean or caearean or cesarian or caearian or "c section" or C-section*):ti,ab,kw (Word variations have been searched) 16875

#65 MeSH descriptor: [Obstetrical Forceps] explode all trees 63

#66 MeSH descriptor: [Vacuum Extraction, Obstetrical] explode all trees 101

#67 ((obstetric* or mechanical or forcep* or vacuum) NEAR/3 (deliver* or extraction* or birth*)):ti,ab,kw (Word variations have been searched) 3879

#68 ("ventouse"):ti,ab,kw (Word variations have been searched) 84

#69 MeSH descriptor: [Labor, Induced] explode all trees 1405

#70 ((labour or labor or Birth or childbirth or deliver*) NEAR/3 (induction or induce*)):ti,ab,kw (Word variations have been searched) 4920

#71 MeSH descriptor: [Seizures] explode all trees 1504

#72 (seizure* or convulsion*):ti,ab,kw (Word variations have been searched) 10881

#73 MeSH descriptor: [Accidental Injuries] explode all trees 6

#74 (injur* or wound* or trauma):ti,ab,kw (Word variations have been searched) 115950

#75 MeSH descriptor: [Hospitalization] explode all trees 22661

#76 (hospitali?ation* or hospital admission* or (HOSPITAL near/33 admitted)):ti,ab,kw (Word variations have been searched) 82428

#77 #71 OR #72 10936

#78 #73 OR #74 115950

#79 #75 OR #76 90159

#80 #77 AND #78 977

#81 #77 AND #79 842

#82 #8 OR #9 OR #10 OR #11 OR #12 OR #13 OR #14 OR #15 OR #16 OR #17 OR #18 OR #19 OR #20 OR #21 OR #22 OR #23 OR #24 OR #25 OR #26 OR #27 OR # 28 OR #29 OR # 30 OR #31 OR #32 OR #33 OR #34 OR #35 OR #36 OR #37 OR #38 OR #39 OR #40 OR #41 OR #42 OR #43 OR #44 OR #45 OR #46 OR #47 OR #48 OR #49 OR #50 OR #51 OR #52 OR #53 OR #54 OR #55 OR #56 OR #57 OR #58 OR #59 OR #60 OR #61 OR #62 OR #63 OR #64 OR #65 OR #66 OR #67 OR #68 OR #69 OR #70 OR #77 OR #78 OR #79 OR #80 OR # 83 1159367

#83 #4 AND #7 AND #82 625

APA PsycInfo <1967 to August Week 1 2023>

1 exp Pregnancy/ 48531

2 exp Expectant Mothers/1023

3 (pregnan* or obstetric* or wom* or gravid*).ab,ti,tw. 375306

4 1 or 2 or 3 387987

5 exp Epilepsy/ 30592

6 (epilepsy or epileptic*).ab,ti,tw. 41010

7 5 or 6 42011

8 exp Pregnancy Outcomes/ 23789

9 exp Obstetrical Complications/ 1782

10 (pregnan* outcome* or adverse pregnan* outcome* or obstetric* complicat* or pregnan* disorder* or obstetric* outcome* or pregnancy complication* or labo?r complication*).ab,ti,tw. 3164

11 (neurodevelopment* or neurodevelopment* disorder or developmental disability* or cognitive development* disorder* or dysparaxia or intellectual disabilit* or pervasive developmental disorder* or behavior* or behavior* disorder* or neurobehavior* disorder* or neurocognitive disorder* or development* regression or developmental coordination disorder* or psychomotor developmental disorder or language delay* or social impairment).ab,ti,tw. 1016168

12 exp Autism Spectrum Disorders/ 55106

13 (autism or autistic).ab,ti,tw. 65712

14 exp Attention Deficit Disorder with Hyperactivity/ 30016

15 (attention deficit hyperactiv* disorder* or ADHD).ab,ti,tw. 39102

16 Sudden unexpected death in epilepsy.ab,ti,tw. 424

17 fetal death.ab,ti,tw. 172

18 birth weight.ab,ti,tw. 6074

19 (IUGR or intra-uterine growth or intrauterine growth or f?etal growth or (f?etus adj3 growth)).ab,ti,tw. 1144

20 still birth.ab,ti,tw. 25

21 ((infant or new born or newborn or neonatal) adj3 mortality).ab,ti,tw. 2111

22 exp Neonatal Intensive Care/ 1979

23 (NICU or neonatal intensive care unit or newborn* intensive care unit).ab,ti,tw. 2565

24 small for gestational age.ab,ti,tw. 741

25 APGAR.ab,ti,tw. 885

26 ((f?etal or f?etus or infant* or neonatal* or newborn* or baby or babies) adj3 hypoglyc?emi*).ab,ti,tw. 74

27 ((newborn* or neonatal* or infant*) adj3 resuscitation).ab,ti,tw. 127

28 exp Spontaneous Abortion/ or exp Induced Abortion/ 3913

29 ((miscarriage* or spontaneous abortion* or habitual abortion* or pregnancy loss* or f?etal loss* or f?etal death* or f?etal demise* or intrauterine loss* or intrauterine death* or intrauterine demise* or abortion*) adj3 (induced or medical*)).ab,ti,tw. 734

30 exp Premature Birth/ 6508

31 ((preterm* or premature) adj3 (birth* or deliver* or infant* or newborn* or baby or babies or neonat*)).ab,ti,tw. 9147

32 exp Gestational Diabetes/ 305

33 (gestational diabete* or "DIABETES IN PREGNANCY").ab,ti,tw. 785

34 exp Preeclampsia/ 134

35 exp Hypertension/ and exp Pregnancy/ 268

36 (eclampsia or preeclampsia or eclamptic or preeclamptic).ab,ti,tw. 745

37 ((HYPERTENSION or HYPERTENSIVE) adj3 PREGNAN*).ab,ti,tw. 311

38 antepartum h?emorrhage.ab,ti,tw. 22

39 POSTPARTUM H?EMORRHAGE.ab,ti,tw. 147

40 exp Postpartum Depression/ 6001

41 ((postpartum or postnatal) adj3 (depression or anxiety)).ab,ti,tw. 6825

42 (premature rupture of membrane* or prelabo?r rupture of membrane*).ab,ti,tw. 65

43 (placenta* adj3 abrupt*).ab,ti,tw. 68

44 ((ectopic or tubal) adj3 pregnan*).ab,ti,tw. 128

45 exp Breast Feeding/ 4180

46 (breast feeding or breast fed).ab,ti,tw. 1777

47 (termination of pregnancy or pregnancy termination).ab,ti,tw. 551

48 (maternal adj3 (death* or motalit*)).ab,ti,tw. 532

49 exp Caesarean Birth/ 438

50 (cesarean or caearean or cesarian or caearian or "c section" or C-section*).ab,ti,tw. 1670

51 ((obstetric* or mechanical or forcep* or vacuum) adj3 (deliver* or extraction* or birth*)).ab,ti,tw. 427

52 ventouse.ab,ti,tw. 3

53 ((labour or labor or Birth or childbirth or deliver*) adj3 (induction or induce*)).ab,ti,tw. 518

54 exp Seizures/ 18289

55 (seizure* or convulsion*).ab,ti,tw. 35261

56 (injur* or wound* or trauma).ab,ti,tw. 182574

57 exp Hospitalization/ 26512

58 (hospitali?ation* or hospital admission* or (HOSPITAL adj3 admitted)).ab,ti,tw. 39597

59 54 or 55 36455

60 57 or 58 52542

61 56 and 59 2578

62 59 and 60 723

63 8 or 9 or 10 or 11 or 12 or 13 or 14 or 15 or 16 or 17 or 18 or 19 or 20 or 21 or 22 or 23 or 24 or 25 or 26 or 27 or 28 or 29 or 30 or 31 or 32 or 33 or 34 or 35 or 36 or 37 or 38 or 39 or 40 or 41 or 42 or 43 or 44 or 45 or 46 or 47 or 48 or 49 or 50 or 51 or 52 or 53 or 59 or 60 or 61 or 62 1180186

64 4 and 7 and 63 1669
