# Supplementary material for: Associations between epileptic seizures in pregnancy and adverse pregnancy outcomes: A systematic review and meta-analysis
Source: PLoS Med. 2025 Oct 31;22(10):e1004580. doi: 10.1371/journal.pmed.1004580 (PMC12578136; doi:10.1371/journal.pmed.1004580)
Supplement: S3 Appendix — (DOCX) [file pmed.1004580.s003.docx]

**S3 Appendix. Study characteristics of included studies**

| Author | Study Title | Year | Country | Study Design | Inclusion criteria | Outcomes |
| --- | --- | --- | --- | --- | --- | --- |
| Jiménez et al. | Epilepsy and pregnancy: Factors associated with epileptic seizures during pregnancy | 2022 | Spain | Prospective cohort study | Pregnant women with epilepsy under follow-up by the epilepsy unit at a tertiary care hospital | Maternal epileptic seizures in pregnancy |
| Hosny et al | Seizure control during pregnancy and postpartum period in women with epilepsy: An Egyptian prospective study | 2023 | Egypt | Prospective cohort study | Pregnant women with known diagnosis epilepsy compliant with their antiseizure medications | Maternal epileptic seizures in pregnancy |
| Meador et al. | Prospective cohort sudy of depression during pregnancy and the postpartum period in women with epilepsy vs control groups | 2022 | USA | Prospective cohort study | Pregnant women ages 14-45 years | Peripartum depression |
| Li et al. | Epilepsy and pregnancy: An audit of specialized care | 2022 | Canada | Retrospective cohort study | 24 weeks or more of gestation, history of at least 3 seizures, definite diagnosis of epilepsy | Miscarriage, induced abortion, maternal epileptic seizures in pregnancy  Congenital anomaly |
| Mehmet et al. | Peripartum haemorrhage and other obstetric and neonatal outcomes in pregnant women with epilepsy: A single-center study | 2021 | Turkey | Retrospective cohort study | WWE who gave birth in the clinic at 24 weeks or more of gestation, followed up in the Neurology Epilepsy Outpatient Clinic with a definite diagnosis of epilepsy and had a history  of at least 3 seizures. | GDM, hypertensive disorders in pregnancy, anaemia, PROM, placental abnormalities, postpartum haemorrhage, uterine atony  Preterm birth, SGA, perinatal death |
| Huang et al. | Clinical characteristics and outcomes in pregnant women with epilepsy | 2020 | China | Retrospective cohort study | Pregnant WWE with 1) in hospital  delivery at Beijing Tiantan Hospital, 2) a diagnosis of epilepsy according  to the 2014 International League Against Epilepsy (ILAE) criteria [5],  3) no history of other chronic disease | Anaemia, hypertensive disorders in pregnancy, PROM, caesarean birth, preterm birth, postpartum haemorrhage |
| Melikova et al. | The impact of maternal epilepsy on delivery and neonatal outcomes | 2019 | Azerbaijian | Prospective cohort study | Patients who were already being followed up for at least 6 months prior to the diagnosis of pregnancy, women enrolled before gestation week 16, and known fetal outcome. | Miscarriage, induced abortion, caesarean birth |
| Trivedia et al. | Spontaneous fetal loss in women with epilepsy: Prospective data from pregnancy registry in India | 2018 | India | Prospective cohort study | Women with epilepsy who were registered in the pre-conception period or during the  first trimester of pregnancy. | Miscarriage |
| Vajda et al | Antiepileptic drug polytherapy in pregnant women with epilepsy | 2018 | Australia | Retrospective cohort study | Pregnant WWE on AEDs | Maternal epileptic seizures in pregnancy |
| Soontornpun et al. | Pregnancy outcomes among women with epilepsy: A retrospective cohort study | 2018 | Thailand | Retrospective cohort study | Pregnant women who attended antenatal care clinic and delivered at Maharaj Nokorn Chiang Mai Hospital. | Hypertensive disorder in pregnancy, caesarean birth, induced labour, antepartum haemorrhage, postpartum haemorrhage  Preterm birth, SGA, low birth weight, apgar score <7 at 5 minutes |
| Watila et al | Seizure occurrence, pregnancy outcome among women with active convulsive epilepsy: One year prospective study | 2015 | Nigeria | Prospective cohort study | Pregnant women with diagnosis of active convulsive epilepsy | Maternal seizures in pregnancy |
| Barroso et al. | Perinatal outcomes from the use of antiepileptic drugs during pregnancy: A case control study | 2014 | Brazil | Retrospective cohort study | Pregnant women with a pre-gestational diagnosis of epilepsy on antiseizure medications during the pregnancy. | Caesarean birth, postpartum haemorrhage  Preterm birth, congenital anomaly, low birth weight perinatal death |
| Abe et al. | Impact of planning of pregnancy in women with epilepsy on seizure control during pregnancy and on maternal and neonatal outcomes | 2013 | Japan | Retrospective cohort study | Pregnant women with epilepsy who were treated at the University of Tsukuba Hospital and Hokkaido University Hospital between 2003 and 2011 | Maternal epileptic seizures in pregnancy |
| Galanti et al. | Postpartum depression in women with epilepsy: Influence of antiepileptic drugs in a prospective study | 2009 | USA | Prospective cohort study | Women presenting to  a tertiary referral center specializing in the management  of perinatal epilepsy during pregnancy or preconception | Peripartum depression |
| Chen et al. | Affect of seizures during gestation on pregnancy outcomes in women with epilepsy | 2009 | Taiwan | Cross sectional study | At least 3 consensus diagnoses of epilepsy or convulsions within 2 years prior to their index delivery | Maternal epileptic seizures in pregnancy,  Low birth weight, preterm birth, SGA |
| Thomas et al. | Cardiac malformations are increased in infants of mothers with epilepsy | 2008 | India | Prospective cohort study | Women with epilepsy enrolled in Kerala Registry of Epilepsy and Pregnancy (KREP) in prepregnancy or early pregnancy period, and live births. | Congenital anomaly |
| Richmond et al. | Epilepsy and pregnancy: An obstetric perspective | 2003 | Canada | Prospective cohort study | Pre-pregnancy diagnosis of epilepsy | Hypertensive disorder in pregnancy, antepartum haemorrhage, induced labour, caesarean birth  Preterm birth, congenital anomaly, perinatal death |
| Majkowska-Zwoli et al. | The rate of and factors associated with delivery by caesarean section among women with epilepsy: Time trend in a single-centre cohort in Mazovia, Poland | 2022 | Poland | Prospective cohort study | Pregnant women with an established diagnosis of epilepsy who gave birth, with enrolment before 16 weeks of gestation, regular once per trimester clinical follow-up, postpartum period visit up to six months | Caesarean birth |
| Sikha Pandey, Ramesh Pandey | Foetal outcome in epileptic women with seizures in pregnancy | 2012 | India | Prospective cohort study | Single births, diagnosis of either epilepsy or convulsions, | Maternal epileptic seizures in pregnancy  Preterm birth, low birth weight, SGA |
| Vajda et al. | Foetal malformations and seizure control: 52 months of data of the Australian Pregnancy Registry | 2006 | Australia | Retrospective cohort study | Pregnant women taking AEDs for epilepsy and women with untreated epilepsy | Miscarriage, congenital anomaly |
| Melikova Shahla, Mammadbayli Aytan | Clinical characteristics, seizure control, and delivery outcomes in pregnant women with focal and generalised epilepsies | 2024 | Azerbaijian | Retrospective cohort study | Pregnant women with epilepsy booked before 16 weeks of gestation and the fetal outcome was known. | maternal seizures during pregnancy, miscarriage, induced labour, PROM, caesarean birth, induced labour  Preterm birth, congenital anomaly |
| Mehmet et al. | Birth outcomes in pregnant women with epilepsy: A nationwide multicenter study from Turkiye | 2023 | Turkey | Prospective cohort study | Pregnant women with epilepsy who were followed up at the neurology outpatient clinics and attended regular follow-up visits. | Congenital anomaly. |
| Vajda et al. | Pregnancy, antiseizure medications and unexplained intrauterine foetal death | 2024 | Australia | Retrospective cohort study | Pregnant women with epilepsy (WWE) who were enrolled in the Raoul Wallenberg Australian Pregnancy Register of Antiepileptic Drugs (APR) between 1999 and the earlier months of 2023. | Perinatal death |
| Olafsson et al. | Pregnancies of Women with Epilepsy: A Population-Based Study in Iceland | 1998 | USA | Retrospective cohort study | Women with epilepsy treated with antiepileptic drugs (AEDs) during pregnancy or during a 5-year period preceding the pregnancy who had live births, products of pregnancies progressing at least through week 28 of gestation. | Congenital anomaly |
| Du et al., | Development and internal validation  of a prognostic model for predicting tonic  clonic seizures during pregnancy in women  with epilepsy | 2024 | China | Retrospective cohort study | Women with epilepsy who were pregnant between January 1, 2010 and December 31, 2020 in ELF URS. | Seizure in pregnancy |
