# Supplementary material for: Associations between epileptic seizures in pregnancy and adverse pregnancy outcomes: A systematic review and meta-analysis
Source: PLoS Med. 2025 Oct 31;22(10):e1004580. doi: 10.1371/journal.pmed.1004580 (PMC12578136; doi:10.1371/journal.pmed.1004580)
Supplement: S4 Appendix — (DOCX) [file pmed.1004580.s004.docx]

**S4 Appendix. Risk of Bias Assessment (Newcastle-Ottawa Scale)**

| **Author** | **Selection** | **Comparability** | **Outcome** | **Total** | **Risk assessment**  **(High, Medium**  **or Low)** |
| --- | --- | --- | --- | --- | --- |
| Jiménez et al., 2022 | 4 | 2 | 3 | 9 | Low |
| Hosny et al, 2023 | 4 | 2 | 3 | 9 | Low |
| Meador et al., 2022 | 4 | 0 | 3 | 7 | High |
| Li et al., 2022 | 4 | 0 | 3 | 7 | High |
| Mehmet et al., 2021 | 4 | 2 | 3 | 9 | Low |
| Huang et al., 2020 | 4 | 2 | 3 | 9 | Low |
| Melikova et al.,2019 | 3 | 2 | 2 | 7 | Medium |
| Trivedia et al.,2018 | 4 | 2 | 2 | 8 | Medium |
| Vajda et al., 2018 | 2 | 0 | 1 | 3 | High |
| Soontornpun et al., 2018 | 4 | 2 | 3 | 9 | Low |
| Watila et al., 2015 | 4 | 2 | 3 | 9 | Low |
| Barroso et al., 2014 | 4 | 0 | 3 | 7 | High |
| Abe et al., 2013 | 4 | 2 | 3 | 9 | Low |
| Galanti et al., 2009 | 2 | 0 | 3 | 5 | High |
| Chen et al.,2009 | 3 | 0 | 3 | 6 | High |
| Thomas et al., 2008 | 4 | 0 | 3 | 7 | High |
| Richmond et al., 2003 | 2 | 0 | 2 | 4 | High |
| Majkowska-Zwoli et al., 2022 | 3 | 1 | 3 | 7 | Medium |
| Sikha and Ramesh, 2012 | 3 | 2 | 3 | 8 | Medium |
| Vajda et al., 2006 | 4 | 0 | 2 | 6 | High |
| Shahla and Aytan, 2024 | 3 | 0 | 3 | 6 | High |
| Mehmet et al. | 4 | 0 | 2 | 6 | High |
| Vajda et al., 2024 | 4 | 0 | 3 | 7 | High |
| Olafsson et al., 1998 | 3 | 1 | 3 | 7 | Medium |
| Du et al., 2024 | 4 | 1 | 3 | 8 | Medium |
