# Supplementary material for: Associations between epileptic seizures in pregnancy and adverse pregnancy outcomes: A systematic review and meta-analysis
Source: PLoS Med. 2025 Oct 31;22(10):e1004580. doi: 10.1371/journal.pmed.1004580 (PMC12578136; doi:10.1371/journal.pmed.1004580)
Supplement: S5 Appendix — (DOCX) [file pmed.1004580.s005.docx]

S5 Appendix. Forest plots


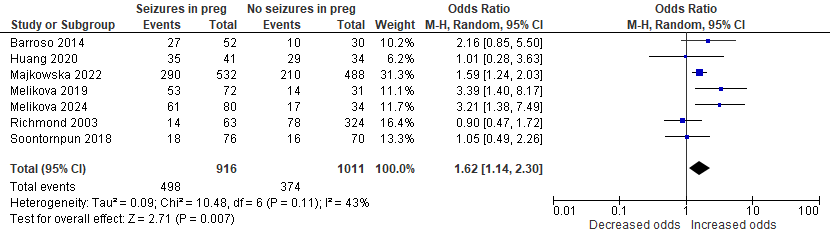


Association between seizures and caesarean birth


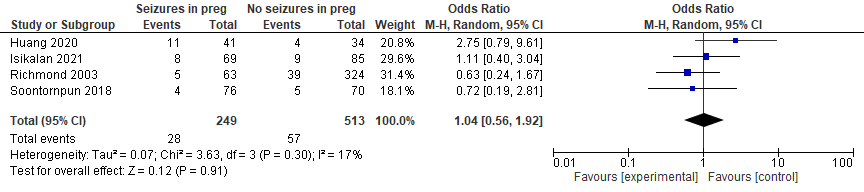


Association between seizures and hypertensive disorder in pregnancy


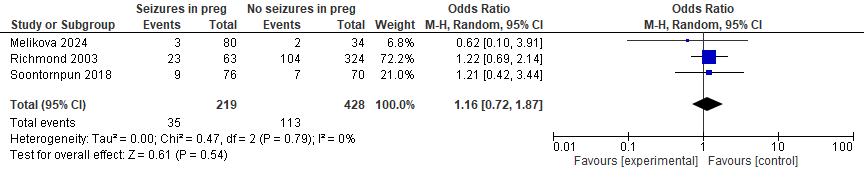


Association between seizures and induced labour


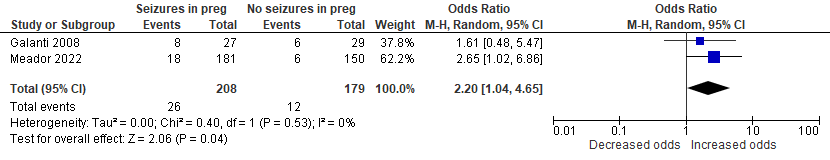


Association between seizures and peripartum depression


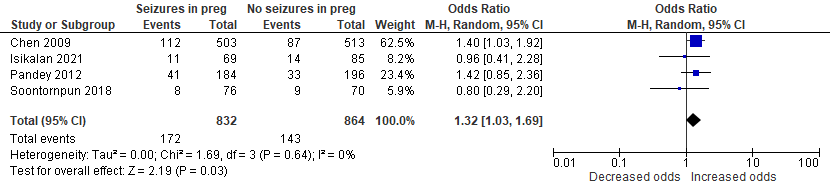


Association between seizures and having small for gestational age babies.


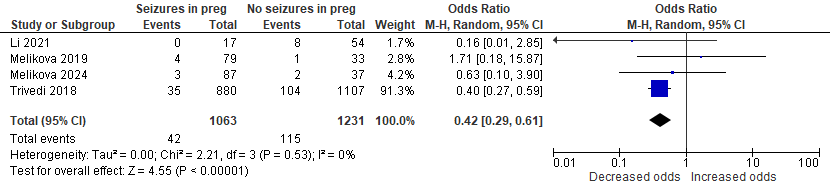


Association between seizures and miscarriage


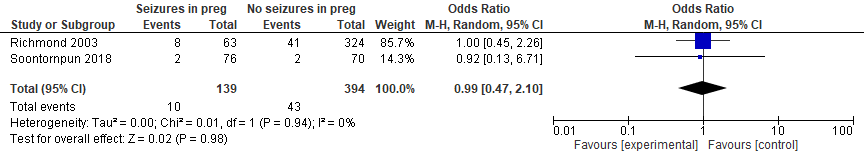


Association between seizures and antepartum haemorrhage


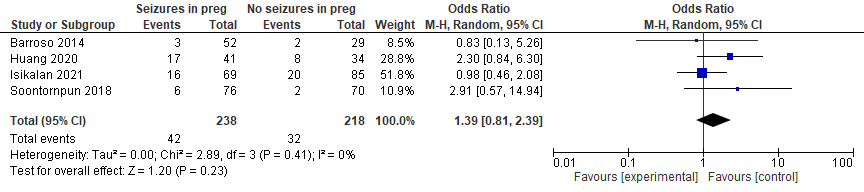


Association between seizures and postpartum haemorrhage


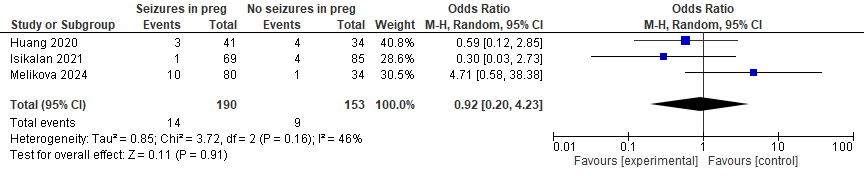


Association between seizures and premature rupture of membrane


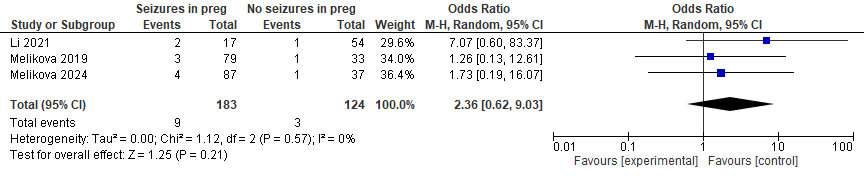


Association between seizures and induced abortion


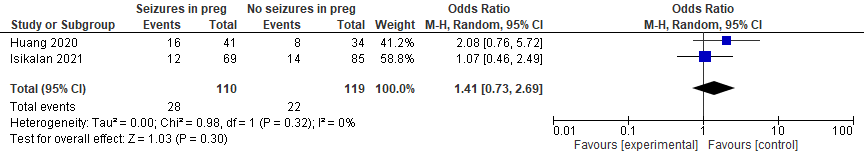


Association between seizures and anaemia


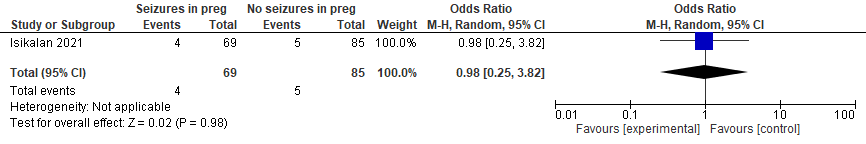


Association between seizures and gestational diabetes mellitus


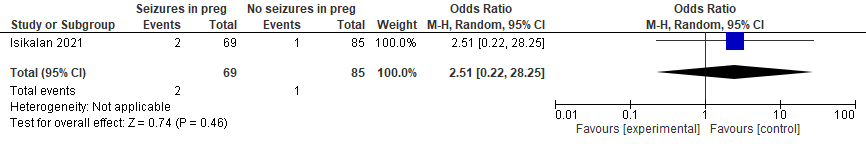


Association between seizures and placenta abnormality


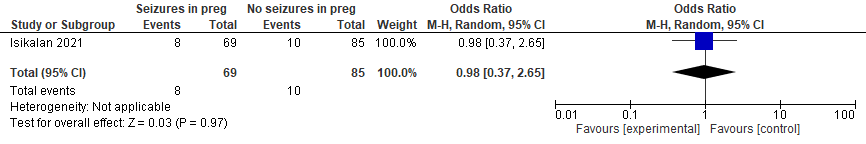


Association between seizures and uterine atony


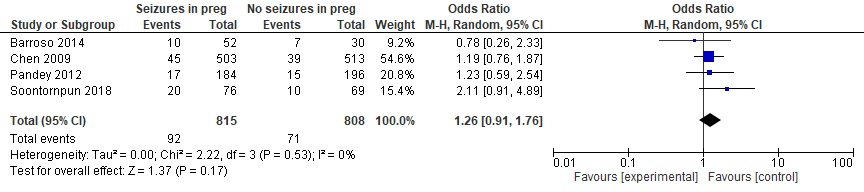


Association between seizures and low birthweight


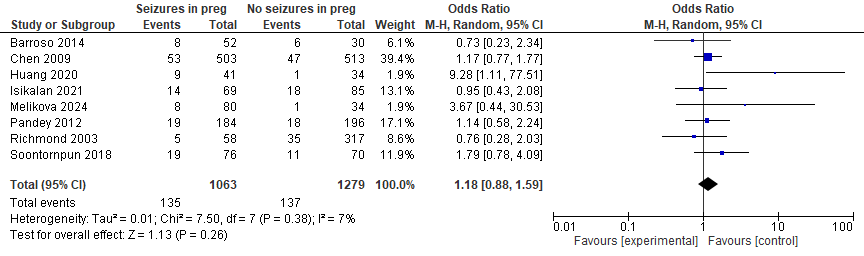


Association between seizures and preterm birth


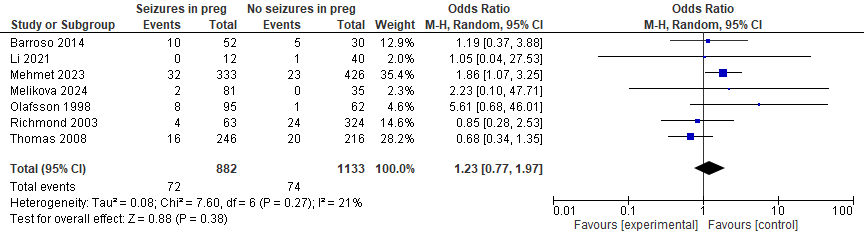


Association between seizures and congenital anomaly


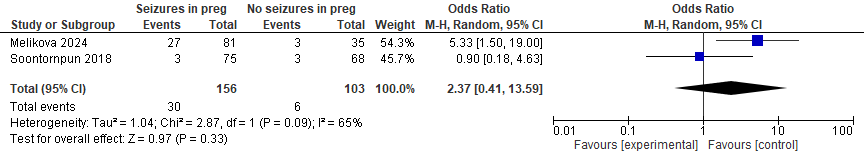


Association between seizures and APGAR <7 at 5 minutes


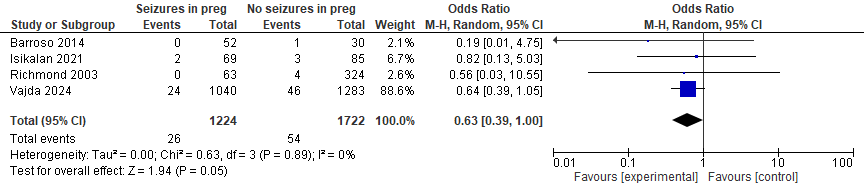


Association between seizures and perinatal death


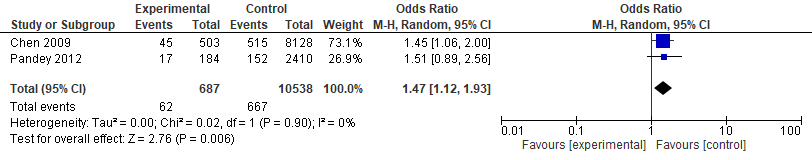


Low birthweight in pregnant women with seizures vs women without epilepsy


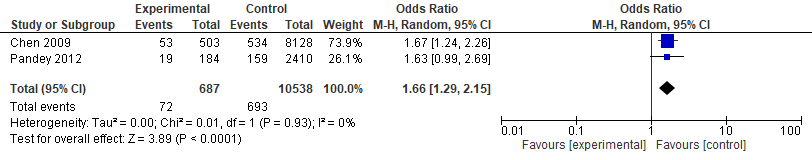


Preterm birth in pregnant women with seizures vs women without epilepsy


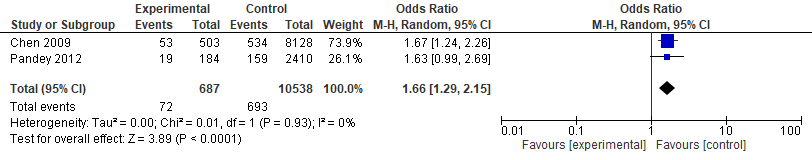


Small for gestational age in pregnant women with seizures vs women without epilepsy


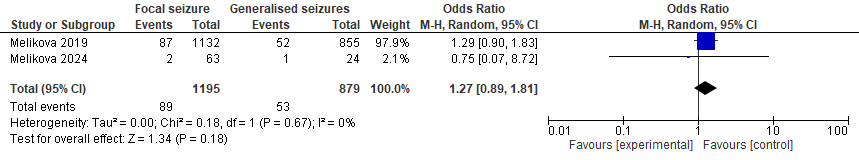


Focal onset vs generalised onset seizures on miscarriage


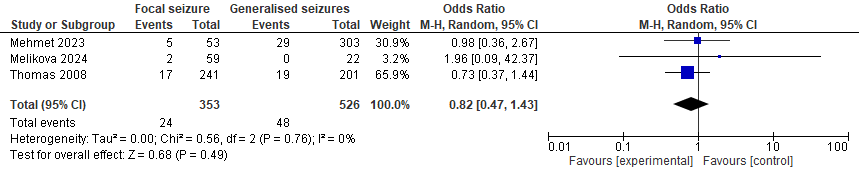


Focal onset vs generalised onset seizures on congenital anomaly


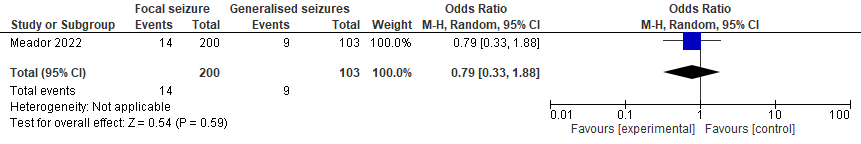


Focal onset vs generalised onset seizures on peripartum depression


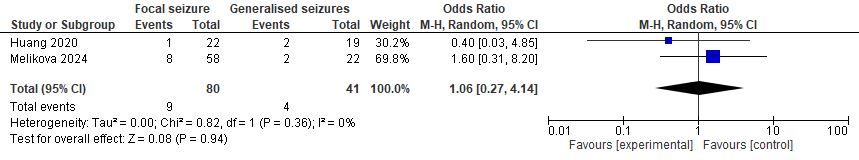


Focal onset vs generalised onset seizures on premature rupture of membrane


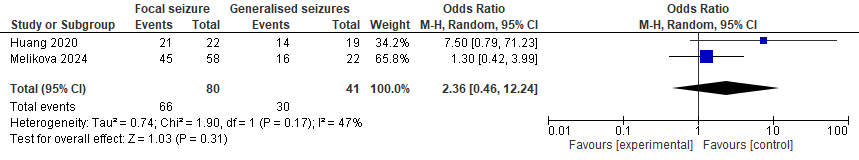


Focal onset vs generalised onset seizures on caesarean birth


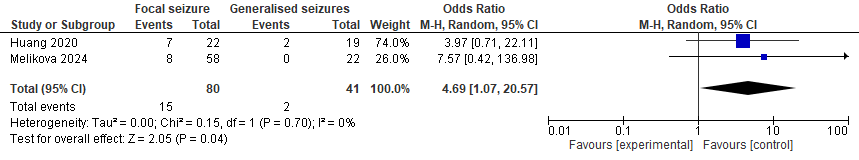


Focal onset vs generalised onset seizures on preterm birth


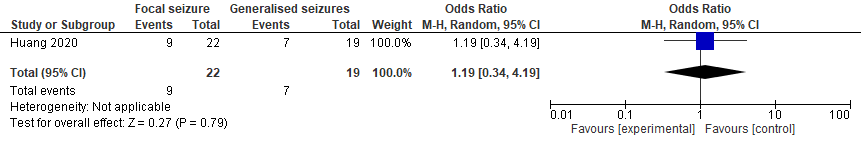


Focal onset vs generalised onset seizures on anaemia


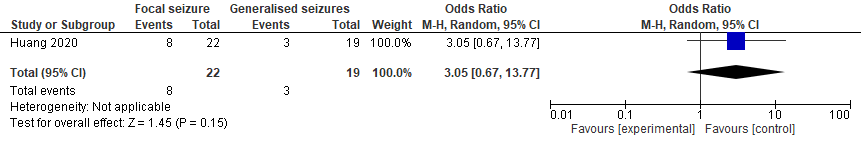


Focal onset vs generalised onset seizures on hypertensive disorder in pregnancy


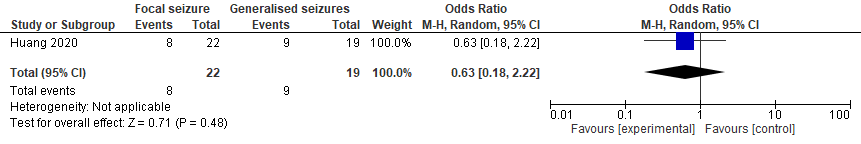


Focal onset vs generalised onset seizures on postpartum haemorrhage


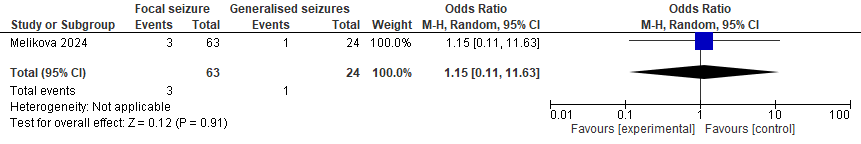


Focal onset vs generalised onset seizures on induced abortion


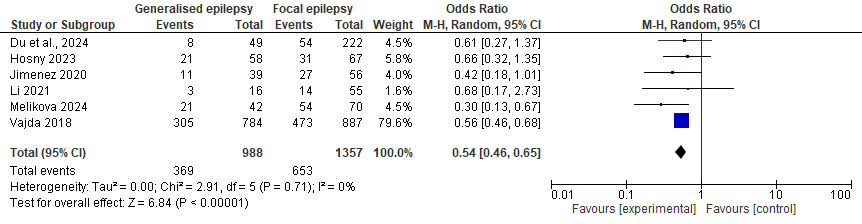
Generalised epilepsy vs focal epilepsy on seizures in pregnancy


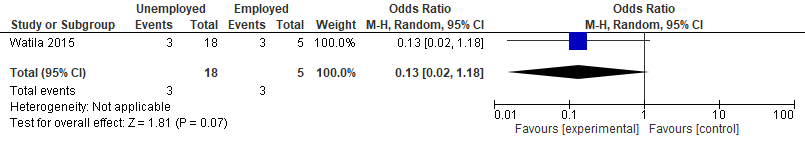
Employment status on seizures in pregnancy


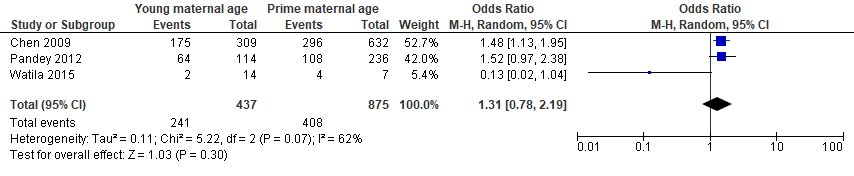
Maternal age ≤ 24 years old vs maternal age 25-34 years old on seizures in pregnancy


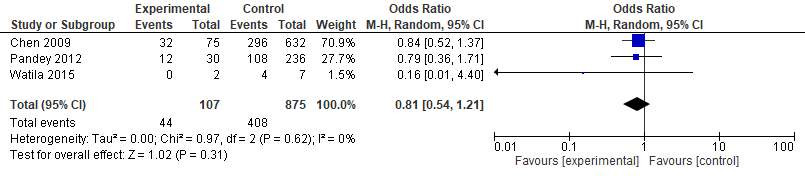
Maternal age ≥ 35 years old vs maternal age 25-34 years old on seizures in pregnancy


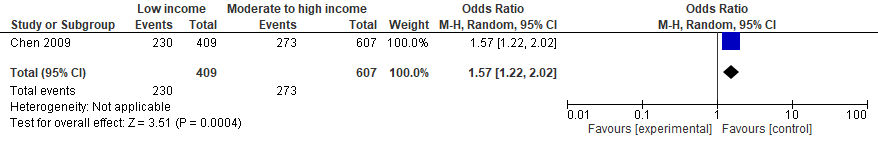
Income status on seizures in pregnancy


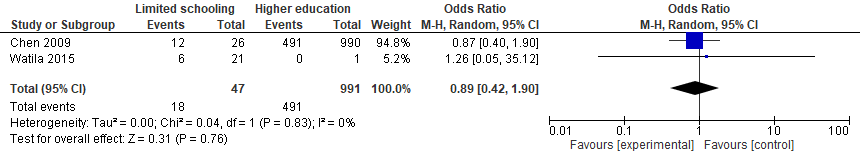
Level of education on seizures in pregnancy


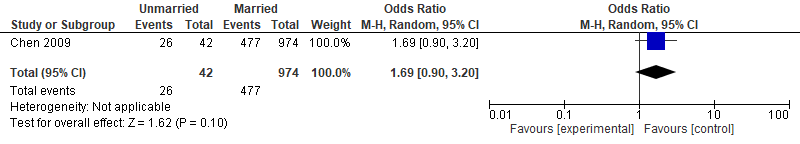
 Marital status on seizures in pregnancy


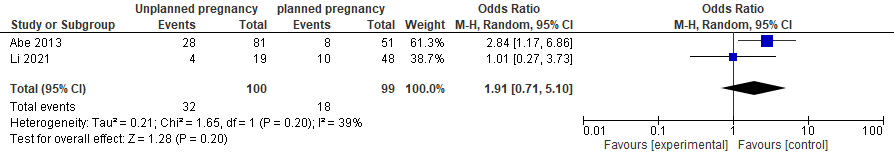
 Planned vs unplanned pregnancy on seizures in pregnancy
