# Supplementary material for: Associations between epileptic seizures in pregnancy and adverse pregnancy outcomes: A systematic review and meta-analysis
Source: PLoS Med. 2025 Oct 31;22(10):e1004580. doi: 10.1371/journal.pmed.1004580 (PMC12578136; doi:10.1371/journal.pmed.1004580)
Supplement: S6 Appendix — (DOCX) [file pmed.1004580.s006.docx]

**Data extraction form**

| **Study** | **Author** | **Comparison** | **Comparison Definition** | **Outcomes** | **Outcome Definition** | **Total No of Women** | **Group 1 with Yes Outcome** | **Group 1 with No Outcome** | **Total Group 1** | **Group 2 with Yes Outcome** | **Group 2 with No Outcome** | **Total Group 2** | **Data missing in Group 1** | **Data missing in Group 2** | **Year** | **Country** | **Country Status** | **Study Design** |
| --- | --- | --- | --- | --- | --- | --- | --- | --- | --- | --- | --- | --- | --- | --- | --- | --- | --- | --- |
|  |  |  |  |  |  |  |  |  |  |  |  |  |  |  |  |  |  |  |
|  |  |  |  |  |  |  |  |  |  |  |  |  |  |  |  |  |  |  |
|  |  |  |  |  |  |  |  |  |  |  |  |  |  |  |  |  |  |  |
|  |  |  |  |  |  |  |  |  |  |  |  |  |  |  |  |  |  |  |
|  |  |  |  |  |  |  |  |  |  |  |  |  |  |  |  |  |  |  |
|  |  |  |  |  |  |  |  |  |  |  |  |  |  |  |  |  |  |  |
|  |  |  |  |  |  |  |  |  |  |  |  |  |  |  |  |  |  |  |
|  |  |  |  |  |  |  |  |  |  |  |  |  |  |  |  |  |  |  |
|  |  |  |  |  |  |  |  |  |  |  |  |  |  |  |  |  |  |  |
|  |  |  |  |  |  |  |  |  |  |  |  |  |  |  |  |  |  |  |
|  |  |  |  |  |  |  |  |  |  |  |  |  |  |  |  |  |  |  |
|  |  |  |  |  |  |  |  |  |  |  |  |  |  |  |  |  |  |  |
|  |  |  |  |  |  |  |  |  |  |  |  |  |  |  |  |  |  |  |
